# Endogenous preparatory control is associated with increased interaction between default mode and dorsal attention networks

**DOI:** 10.1162/imag_a_00124

**Published:** 2024-04-08

**Authors:** Max K. Egan, Cyril Costines, Mark D’Esposito, Sepideh Sadaghiani

**Affiliations:** University of Illinois at Urbana-Champaign, Champaign, IL, United States; Beckman Institute for Advanced Science and Technology, Urbana, IL, United States; Helen Wills Neuroscience Institute, University of California, Berkeley, Berkeley, CA, United States

**Keywords:** cognitive control, intrinsic functional connectivity networks, fMRI, network interactions

## Abstract

It is increasingly recognized that cognitive control requires integration across large-scale brain networks anchored in frontal and parietal cortices. While the functional role of individual networks has been studied extensively, their cross-network interactions in the service of cognitive control are poorly understood. Beyond in-the-moment regulation of goal-relevant information processing (e.g., of sensory information), cognitive control encompasses preparatory processes in anticipation of upcoming stimuli and actions. Such preparatory control is often endogenous, that is, it is based on internal representations without relying on external cues or events. Here, we assessed network interactions that support such endogenously driven preparatory control. We recorded fMRI (N = 25) during a perceptual decision task with highly variable intertrial intervals. In half of the blocks, trial onset was cued, while in the remaining blocks, participants maintained readiness without relying on cues. We studied endogenous preparatory control in the intertrial period preceding uncued (vs. cued) trials. Behavioral outcomes confirmed heavier cognitive control demands in the uncued condition. Endogenous preparatory control was associated with increased activity of the dorsal attention network (DAN). This contrasted with in-the-moment control over stimulus-response processing during the trial itself, which was supported foremost by the right-hemispheric fronto-parietal network (FPN). Cross-network interactions were strengthened exclusively during endogenous preparatory control; the default mode network (DMN) showed more positive connectivity with the DAN and to a lesser degree the cingulo-opercular network (CON). Our results demonstrate that cross-networks interactions are particularly important for endogenously driven preparatory control. They further suggest that the DMN may be implicated in internally harnessing resources for cognitive control. This notion extends the DMN’s known role in internally-oriented processing to the domain of cognitive control when preparation cannot be aided by external events.

## 1 Introduction

Cognitive control consists of several top-down modulatory functions that enable goal-directed behavior (Hommel et al., 2019;Lindsay, 2020;Oken et al., 2006;Posner & Petersen, 1990), comprising both the in-the-moment modulation over processing of incoming stimulus information as well as the maintenance of preparatory processes. Such preparatory control comprises a variety of functions, such as maintaining task set and goals in working memory, and keeping at the ready the neural recourses required for processing upcoming stimuli in accordance with those goals (Forstmann et al., 2005). While preparatory control processes*can*be initiated by an external event such as a warning signal, they are often engaged in the absence of such proximal cues or sensory events in everyday life. For example, we may be actively waiting for the ring tone of a scheduled online meeting, maintaining readiness to take the incoming call. In the current work, we refer to this aspect of real-life cognitive control as*endogenously driven*preparatory control. Endogenous neural processes denote those that are primarily based on internal representations, contrasting exogenous processes that involve foremost incoming environmental information (Forstmann et al., 2005). More specifically, we conceptualize endogenously driven preparatory control as the internal (i.e., not stimulus/event-initiated) maintenance of readiness of task-relevant cognitive faculties. In other words, this concept is an umbrella term that encompasses various top-down control functions occurring prior to and in anticipation of goal-directed processing of stimuli, decisions, and actions, and is maintained without external initiation.

In terms of neural processes, it is increasingly recognized that cognitive control require integration*across*large-scale brain networks, each comprising a specific set of distributed regions anchored in frontal and parietal cortices (Menon & D’Esposito, 2022). While much progress has been made in delineating the individual large-scale brain networks involved in cognitive control (Corbetta & Shulman, 2002;Menon & D’Esposito, 2022;Sadaghiani & D’Esposito, 2015;Vossel et al., 2014), their cross-network*interactions*in the service of cognitive control are poorly understood.

Specifically, several canonical Intrinsic Connectivity Networks (ICNs) have been consistently observed in neuroimaging studies as contributors to cognitive control functions. These include the Cingulo-Opercular Network (CON), the lateral Fronto-Parietal Network (FPN), and the Dorsal Attention Network (DAN). While the DAN and FPN act in a largely phasic manner, the CON is thought to maintain control more tonically (Dosenbach et al., 2006;Sadaghiani & Kleinschmidt, 2016). Specifically, the FPN is thought to support phasic and rapid moment-to-moment adjustment of cognitive control such as in exogenously triggered initiation of control (Dosenbach et al., 2007) or of phasic alertness (Sadaghiani et al., 2012), adapting after errors (Dosenbach et al., 2007) and repeated rapid task switching (Seeley et al., 2007). The DAN is known to enable selective attention especially in visual and spatial domains (Corbetta & Shulman, 2002); it is thought to maintain spatial priority maps to guide overt or covert manipulation of attention in space and visual working memory. Conversely, the CON has been implicated in sustained alertness (Coste & Kleinschmidt, 2016;Sadaghiani & D’Esposito, 2015;Sadaghiani et al., 2009,^2010^) and continuous task-set maintenance (Dosenbach et al., 2006). We note that the CON may at least partially overlap with the so-called Salience Network (which in turn overlaps functionally and spatially with the Ventral Attention Network) (Seeley et al., 2007;Sridharan et al., 2008). Contrasting the sustained control function of the CON, the Salience Network is thought to engage transiently in the service of detection and evaluation of salient external and internal signals (Menon & Uddin, 2010). While the Salience Network is thought to kick off control processes (maintained by other networks) when salient events occur, it is not thought to contribute substantially to*preparatory*control processes. Therefore, we do not expect salience-related activity in our contrasts that aim to isolate preparatory control, but rather anticipate seeing sustained control processes—the kind associated with the CON—throughout of the task. Additionally, a fourth network, the DMN, commonly decreases in activation in most task settings, yet plays an important role in cognitive control processes. Some of these processes overlap with those that activate the above-described control networks, such as task switching (Crittenden et al., 2015), and covert shifts in spatial attention (Arsenault et al., 2018), while others such as self-referential attention (Bressler & Menon, 2010) are more unique to the DMN. Due to this engagement in some control tasks, more recent accounts consider the DMN among control-related networks (Menon & D’Esposito, 2022).

Activity in the regions within each of the above-described control-related ICNs changes in concert in response to task demands (Braun et al., 2015;Cole et al., 2013;Corbetta & Shulman, 2002;Dixon et al., 2018;Fornito et al., 2012;Menon & D’Esposito, 2022;Spreng et al., 2010) as well as spontaneously during task-free rest (Di & Biswal, 2014;Fox et al., 2005;Spreng et al., 2013). The consistent change in activity levels among the nodes of each of these networks supports the notion that each network represents a dissociable functional unit (Menon & D’Esposito, 2022). Critically however, these networks do not work in isolation but instead achieve cognitive control through a dynamic interplay of cross-network segregation and integration (Cohen et al., 2014;Gratton et al., 2018;Kitzbichler et al., 2011;Liang et al., 2016;Sadaghiani et al., 2015;Shine et al., 2016). In fact, some early frameworks that delineated the involvement of individual ICNs in different components of cognitive control (Corbetta & Shulman, 2002) have given way to more recent conceptualization of increasing and decreasing cross-ICN interactions (Cocchi et al., 2013;Corbetta et al., 2008). Accordingly, network-based neuroimaging has recently embarked on characterizing between-ICN versus within-ICN connectivity under conditions of heightened cognitive control (Cocuzza et al., 2020). Yet, the functional role of cross-ICN interactions specifically in endogenously driven preparatory control remains largely unknown.

In the context of laboratory experiments, investigating endogenously driven preparation poses challenges due to the necessity of manipulating experimental conditions. This manipulation is most often achieved by using external events, which requires processing of incoming information and may thus obfuscate the isolation of endogenous control processes. Examples of external signals or events used to guide “endogenous” preparatory control within the experiment include cues that direct the subject’s attention towards the upcoming stimulus (Braver, 2012;Coull et al., 2000;Posner & Petersen, 1990) or stimuli that inform the subject which task rules to follow on the upcoming trial(s) (Braver & Bongiolatti, 2002;Forstmann et al., 2005) either in the form of direct instruction or feedback (e.g., motivating a shift to a correct rule set). All these approaches still rely on the existence of external inputs that provide information on a trial-by-trial basis. However, as discussed above, preparatory control in everyday life does not always result from external changes but can be based primarily on internal representations.

To target such endogenously driven maintenance of task goals and readiness, our experimental manipulations apply to all trials of a given block such that participants can internally sustain control processes independently of incoming information. This approach parallels prior paradigms that modulate control demands in a global (run- or block-wise) rather than local (trial-by-trial) manner (e.g.,Braver et al., 2021). Critically however, neuroimaging analyses of such prior studies nevertheless have been largely driven by stimulus-evoked activity. As canonical examples, N-Back, AX-CPT, and Stroop paradigms require endogenous maintenance of goal-related readiness that is experimentally manipulated over blocks/runs, for example, by changing the probability of different trial types across blocks/runs. Yet, neuroimaging investigations typically contrast blocks/runs either in terms of neural responses to certain trial types of interest (targets, incongruents, etc.) or to all trials of the block/run. Contrarily, to better isolate endogenously driven preparatory control, we focus on the period*preceding*the trial, in particular when the trial does*not*contain a prestimulus cue to aid readiness.

**Fig. 1.**
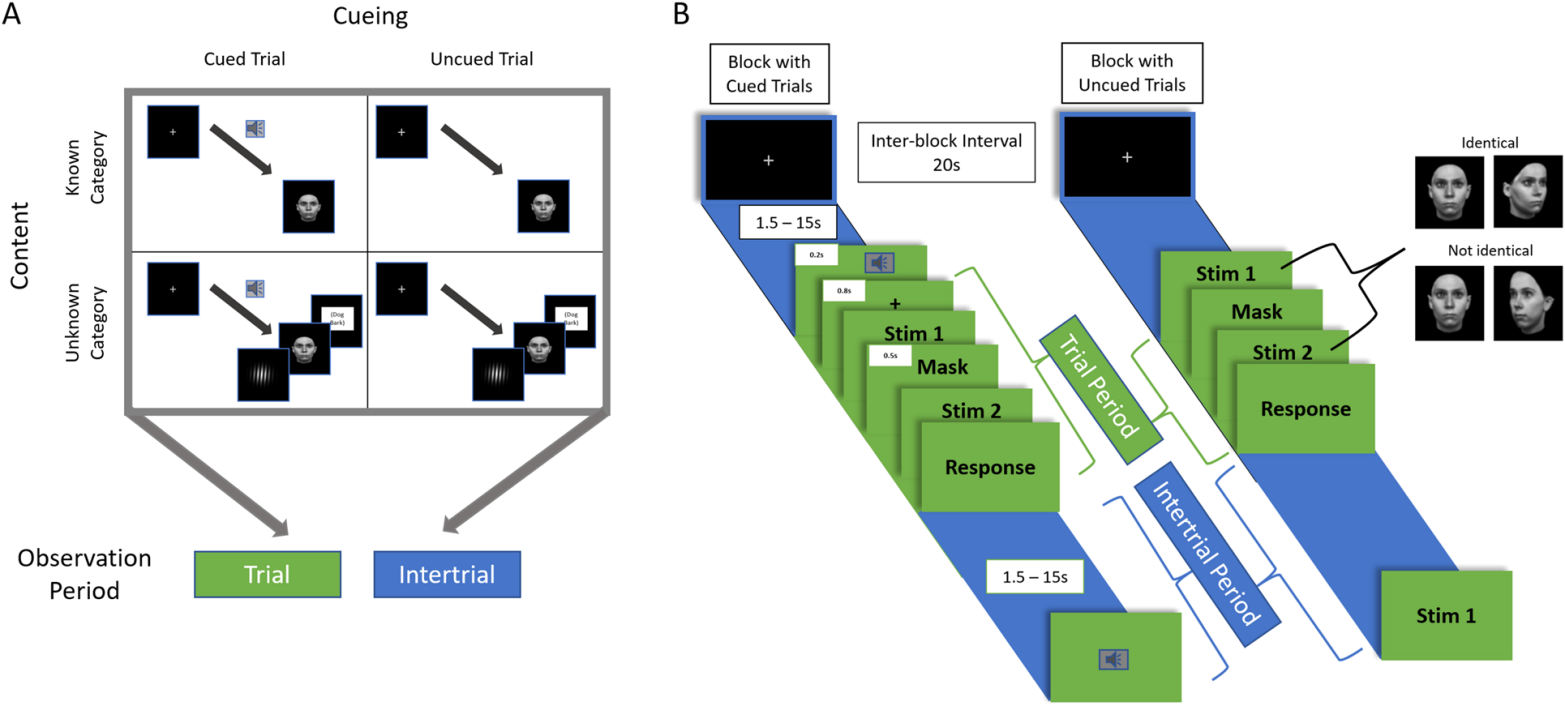
Experimental conditions and design. (A) The 2x2 conditions of the experiment consisting of the factors “Cueing” and “Content,” manipulated over blocks. Experimental blocks within each run were either Cued or Uncued, with all stimuli within the block being preceded by the auditory warning cue or having no cue, respectively. Blocks were further split by “Content” in which all stimulus categories were known beforehand (only faces), or unknown (randomly chosen). All analyses in this study were performed for two different time epochs. The Intertrial period consisted of the time from fixation onset after a given trial up to the presentation of either the cue or the uncued stimulus on the next trial. The Trial period consisted of the time period comprising cue (on cued trials), stimuli, and response. The primary contrast of interest was the difference between Uncued and Cued conditions during the Intertrial period, requiring heightened endogenously driven preparatory control. (B) Time course of the experiment. The blue sections represent the Intertrial period leading up until the trial itself, and the green section encompasses the Trial period. Note that we chose to maximize behavioral cue-efficacy by keeping the cue 100% informative in indicating the imminent onset of an upcoming trial and not to jitter cue-to-stimulus delay, and did not aim at dissociating cue-related and other processes during the Trial.

Specifically, in our paradigm, subjects responded to a stimulus-pair presented sequentially on each trial, indicating whether the second stimulus is the same as the first (Fig. 1). Trials occurred at long and highly varied inter-trial intervals. They were embedded in blocks that either do or do not contain auditory cueing just prior to stimulus onset, that is, a warning signal that the trial is about to start. Because stimuli were brief and difficult to dissociate, successful processing during uncued blocks required goal-directed (task-specific) readiness during the inter-trial intervals, well isolating endogenously driven preparatory control. Contrarily, during cued blocks, the cue was 100% informative with a fixed duration between cue and stimulus onset. Subjects could thus rely on the external cues to engage control processes when needed, minimizing the need for preparatory control prior to the cue.

We further asked whether endogenously driven preparation depends on the degree to which stimulus content is known. Knowing the upcoming stimulus type informs about which specific “lower-order” brain areas are task-relevant, possibly enabling more specific preparatory regulation of stimulus-related areas (e.g., fusiform face area engagement when the upcoming stimulus is known to be a face). To this end, we used two different stimulus conditions across blocks, one which comprises a single stimulus category within a single sensory modality (face images) and another condition randomly drawn from seven categories across visual and auditory sensory modalities. Adopting this 2x2 design that manipulated cueing and stimulus content across blocks, we investigated how interactions among cognitive control ICNs enable endogenously driven preparatory control during the intertrial period.

## 2 Methods

### 2.1 Human subjects

Twenty-five subjects (13 female) with average age of 21.6 years (±2.2, range 18-27) underwent fMRI recording. All subjects were right-handed, and had no history of neurological or psychiatric disorders. The study was approved by the Institutional Review Board of the University of California, Berkeley, and all subjects gave written informed consent according to the approved procedures.

The sample size was chosen based on group-level effect sizes that we observed previously in a comparable study with within-subject design. Specifically, our prior study (Sadaghiani & D’Esposito, 2015) aimed at dissociating the role of the control-related ICNs, albeit across other cognitive control processes (specifically high vs. low alertness). This prior study used the same scanner and fMRI sequence and was recruited from the same community with comparable demographics as the current study. With a sample size of 20, the prior study demonstrated significant differences in the activation (paired*t*_19_= 3.53, corresponding to Cohen’s*d*= 0.79 at*r=0.5*between paired measures) and connectivity (paired*t*_19_= 3.87, Cohen’s*d*= 0.87) of the targeted cognitive-control ICN. To detect an effect size of*d*= 0.79 with a power of 0.95, a sample size of 19 is required. We therefore chose to meet (and slightly exceed) the sample size of the prior study.

### 2.2 Experimental paradigm

On each trial, subjects were presented sequentially with a stimulus pair and were instructed to respond if the second stimulus in the pair was the same as the first stimulus (stimulus details provided below). In each of four 10.5 min runs, every subject was presented with six blocks of eight trials each. Blocks were separated by inter-block intervals of 20 s during which the subjects viewed a central fixation cross.

Blocks were manipulated across two different factors: a “Cueing” factor in which the onset of the trial was or was not preceded by an auditory warning cue of 200 ms duration using a pure tone of 275 Hz, and a “Content” factor in which the trial content was either known or unknown. The warning cue indicated the onset of a trial with 100% reliability, that is, the occurrence of this sound was*always*followed by stimuli after a fixed delay of 1 s from cue onset. Blocks with known content consisted of face stimuli only (cf. below). Blocks of unknown content consisted of stimuli drawn unpredictably from seven possible categories (cf. below). Because subjects were not explicitly informed about the task condition, all blocks of known and respectively unknown content were presented in separate runs, allowing the subject to rapidly infer the condition at the beginning of the run. This separation also provided a sufficient number of stimulus repetitions to adequately model brain responses to each stimulus category in the unknown content runs. Estimability was further supported by limiting—without the subject’s awareness—each run of unknown content to four of seven stimulus categories (human faces plus three other randomly selected categories). In total, every subject completed six blocks of each factor-level pairing (e.g., cued and known content, cued and unknown content, uncued and known content, and uncued and unknown content).

The seven stimulus categories comprised visual (human faces, gabor patterns, visual motion) or auditory (human voices, animal sounds, pitch detection, auditory motion) content:

- Human face stimuli were drawn from a face database of the Max-Planck Institute for Biological Cybernetics’ Department of Human Perception, Cognition, and Perception (Wallraven & Bülthoff, 2001). The chosen database provided highly realistic, computer-generated faces that were precisely controlled for low-level visual features such as illumination, while providing numerous viewpoints (face orientations) for each face. We used 20 male and 20 female faces with neutral facial expression. The black-and-white face images were presented for 100 ms, with both the first and second face presented randomly at either forward-facing orientation or a 30-degree rotation to either the left or right. Different orientations were used in order to target high-level face processing rather than low-level visual features; the subject was tasked with deciding if the identity of the person matched between the two stimuli irrespective of the visual angle.
- Gabor patches were presented at one of five orientation levels in increments of 5-degree steps (35, 40, 45, 50, 55°) for 100 ms. The subject was tasked with deciding if the orientation matched between the two stimuli.
- Visual motion stimuli consisted of random dot kinematograms containing 100 dots that moved horizontally left- or right-ward with 80% coherence at a spend of 4.5 degrees/second, and were presented for 300ms. The subject was tasked with deciding if the motion direction was the same or different across the two stimuli.
- For voice recognition, human voices were chosen from the internet and consisted of three male individuals and three female individuals. Each speaker read five complete sentences that were cut in half to make a total of 10 sound clips from each person. The voice segments had a mean duration of 990ms with a minimum duration of 570 ms and maximum of 1.32 s. The subject was tasked with deciding if the same person was speaking across the stimulus pair.
- Animal sounds were taken from the internet, with three different sounds presented from each of 10 animals (dogs, cats, ducks, seagulls, frogs, yellowthroats, finches, sheep, cows, donkeys). The animal sounds had a mean duration of 980 ms with a minimum duration of 290 ms and maximum of 1.1 s. The subject was tasked with deciding if the animal species was the same across the stimulus pair.
- Auditory pitch detection involved sinusoidal sounds on each trial chosen from 11 sounds and presented for 200 ms. The possible sounds comprised 440 Hz (C4 frequency) and 10 additional sounds increasing in quarter tone steps on a 12-tone equal temperament scale. On identical trials, the same sound was played for both the first and second stimulus. On non-identical trials, the two sounds were a quarter tone apart. The subject was tasked with deciding if the pitch was the same across the two stimuli.
- Auditory motion consisted of a sinusoidal sound of 440 Hz, perceived as moving left- or right-ward over a 200 ms duration as a result of binaural volume difference (volume decreasing linearly in one ear and increasing in the other). The subject was tasked with deciding if the motion direction was the same or different across the two stimuli.

The stimulus pairs were presented 500 ms apart from each other with a mask present during the inter-stimulus interval (a circle filled with “static” white dots for visual trials, and white noise for auditory trials). The mask minimized the ability to accomplish the same/different decision by simple change detection. During the auditory trials, the fixation cross was present during the mask period. The inter-trial interval between stimuli pairs was highly variable, randomly varying between 1.5-15 s. Specifically, each block contained eight random selections (without replacement) from the following ITI durations: 1.5 s, 3 s, 4.5 s, 6 s, 7.5 s, 9 s, 10.5 s, 12 s, 13.5 s, and 15 s.

Two different periods were investigated. The “Intertrial” period consisted of the entire time of fixation following one trial up until onset of the next trial—representing the period of endogenous preparatory control. The “Trial” period consisted of the entire period from cue onset (on cued trials) or first stimulus onset (on uncued trials) until the subject’s response, where control included in-the-moment modulation of external information. Note that the paradigm was not designed to dissociate different processes*during*the Trial period, such as cue-evoked versus stimulus-evoked activity, or to speak to the endogenous/exogenous nature thereof. Such dissociations are beyond the scope of our interest specifically in endogenous preparatory control and would have required design choices that may jeopardize the behavioral efficacy of the cue and the ensuing impact on the Uncued versus Cued distinction in the Intertrial period of interest.

### 2.3 MRI data collection

Imaging data were collected on a whole-body 3-T Siemens MAGNETOM Trio MRI scanner using a 12-channel head coil. Subjects performed the above-detailed task in a total of four runs of 315 volumes (10.5 min) each. Whole-brain functional images were acquired using the following parameters: T2*-weighted echo planar imaging sequence with TR = 2000 ms, TE = 28 ms, flip angle = 78°, 3 × 3 mm in-plane resolution, 210 × 210 mm field of view, and 32 3-mm-thick oblique transversal slices with 0.3 mm interslice gap in descending contiguous order. Scalp fat signal was minimized using fat saturation. Structural images were acquired using a magnetization-prepared rapid gradient echo T1-weighted sequence with the following parameters: TR = 2300 ms, TE = 2.98 ms, flip angle = 9°, and 1 × 1 × 1 mm voxels.

### 2.4 fMRI preprocessing and handling of head motion

The following preprocessing steps were performed in SPM12 in successive order: slice-timing correction, re-alignment, normalization to MNI space, and smoothing with a 6 mm^3^kernel. To assess that head motion did not systematically differ across the contrasts of interest, framewise displacement (FD) values were calculated for each condition for every subject (mean ± standard error of FD across all subjects of 0.1489 ± 0.0092, 0.1444 ± 0.0077, 0.1448 ± 0.0083, and 0.1481 ± 0.0095 for the Cued Known, Uncued Known, Cued Unknown, and Uncued Unknown conditions, respectively). A repeated-measures ANOVA using these FD values showed that there were no systematic head motion differences across the main effects of Cueing (F_1,24_= 0.035,*p*= 0.854), Content (F_1,24_= 0.001,*p*= 0.973), or the interaction between them (F_1,24_= 1.965,*p*= 0.174).

### 2.5 Independent ICA-derived network maps

All comparisons to ICNs used the ICN maps provided in the Independent Component Analysis (ICA)-derived atlas of the FIND lab from an independent cohort (Shirer et al., 2012). Specifically, the spatial independent components used from the atlas are the (dorsal) default mode network (DMN), visuospatial network (labeled here as the dorsal attention network, DAN), left and right fronto-parietal executive control networks (lFPN/rFPN, respectively), and anterior salience/cingulo-opercular network (CON). Beyond the full map for each independent component (ICN), the atlas provides the map of each individual region of the ICNs, which we used for the connectome-wide PPI analysis (cf.Fig. 6).

### 2.6 Task-based activation analysis

Group-level analyses were performed using general linear models (GLM) in SPM12. The first-level GLM for each subject included regressors for each stimulus category, seven for cued and seven for uncued trials. Note that by design, the entire trial was modeled as one event (single impulse response convolved with the canonical hemodynamic response function), and the experiment was*not*aimed at separating post-cue activity from other trial-related processes. Crucially, we further estimated activity during the Intertrial period (against inter-block intervals serving as implicit baseline), with one regressor for Intertrial periods during cued blocks, and one for uncued blocks. Six rigid body head motion parameters were added as nuisance regressors.

Contrasts assessed the effects of Cueing, and its interaction with Content, in both the Intertrial and Trial time periods. Additionally, we compared all the above-described Trial and Intertrial regressors (irrespective of condition) against implicit baseline (i.e., inter-block interval) to confirm the expected engagement of the task-positive ICNs during the task blocks. We further investigated the reverse contrast to confirm higher engagement of the DMN during inter-block versus active task block periods.

To assess task-based activation overlap with the ICA-derived network maps, the total number of voxels within the junction of each network map and the space of all significant voxels in the contrast of interest was calculated, and then divided by the total number of significant voxels in the contrast of interest.

### 2.7 Seed-based PPI analysis

Context-dependent connectivity was investigated using psychophysiological interaction (PPI) analysis in SPM12. For every subject, two PPI models were generated. For each of the two models, the seed volume was derived based on results of the task-based activation analysis described above. Specifically, the first model defined the seed volume as the resting-state ICN-derived DAN, which was adjusted to individual subjects by masking with all voxels passing*p*< 0.05 (uncorrected) in the Uncued Intertrial > Cued Intertrial contrast at single-subject level. For this PPI model, the context-dependent contrast was the Uncued Intertrial > Cued Intertrial contrast. The second model defined the seed volume as the ICN-derived rFPN adjusted to individual subjects by masking with all voxels passing*p*< 0.05 (uncorrected) in the Uncued Trial > Cued Trial contrast. The context-dependent contrast in this PPI model was Uncued Trial > Cued Trial contrast. The choice of ICA-derived network mask (DAN and rFPN, respectively) was informed by the outcome of the contrasts of interest in the preceding task-based activation analysis (seeResults).

### 2.8 Connectome-wide PPI analysis

To investigate whole-brain context-dependent connectivity, a PPI model was generated separately for every individual region within the DMN, DAN, lFPN, rFPN, and CON of the ICA atlas, with each region serving as a seed, for a total of 39 seeds for each subject. All 39 regions also served as “target” regions of each PPI; for each seed, the parameter estimates of the context-dependent correlation (i.e., PPI regressor) were averages over all voxels within each of the 39 regions. This procedure resulted in a 39 seed x 39 target connectivity matrix. Each row in the matrix represents the seed region from which the PPI was generated, and each column represents the estimated context-dependent co-activation of the target regions with the PPI seed. As a result, all connectivity matrices are inherently directional, that is, asymmetrical (which however does not reflect causality). For every subject, two sets of connectivity matrices were generated, one set for the Intertrial and one for the Trial epochs. Each of these sets contained four separate matrices for each block type of the experiment—cued with known content, uncued with known content, cued with unknown content, and uncued with unknown content. In other words, each condition of the factorial design was used for a separate context-dependent contrast.

Separately for Intertrial and Trial epochs, the four PPI connectivity matrices of the four conditions were entered into a repeated-measures*F*-test. Specifically, the*F*-test design matrix contained three contrasts—one for the factor of Cueing, one for the factor of Content, and the interaction term between them. After thresholding each element (connection) of the matrix at*F*_(1,24)_>9.55 (*p*< 0.005), Network-Based Statistics (NBS) (Zalesky, 2010) was applied to identify clusters of interconnected connections that show significant effects in the respective contrast. NBS is a non-parametric method that deals with the multiple comparisons problem (multitude of connections) on a connectivity matrix (graph) by identifying clusters of connections in topological space whose size surpasses chance level while controlling the family-wise error rate. To this end, we used the Brain Connectivity Toolbox in MATLAB® 2020a with the directed NBS extension due to the asymmetric nature of the matrices. NBS was applied to every contrast (Cueing, Content, and their interaction) for both the Intertrial and Trial epochs.

## 3 Results

### 3.1 Behavioral outcomes

In this study, we aimed to identify brain networks and network interactions that support endogenous preparatory control, specifically of task goals and the readiness to process task-relevant sensory information, when no cue is available to initiate such control. Our central condition of interest was thus the Uncued condition (compared to the Cued condition) during the anticipatory Intertrial period. For comparison, we further assessed how the lack of cues (Uncued>Cued) impacted in-the-moment processes that engage upon availability of external information*during*the trials. After investigating behavioral effects, we first characterize the activation levels of each network before moving to their connectivity profile and inter-network interactions.

**Fig. 2.**
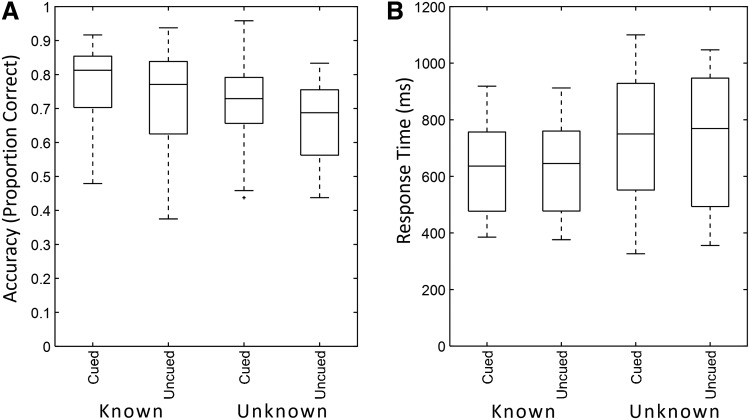
Behavioral results. Accuracy and response times across participants (median and 25/75^th^percentiles) shown separately for the conditions of Cueing and Content factors. (A) Accuracy decreased during the Uncued condition, and this cueing effect was present for both the Known and Unknown Content conditions (no significant interactions). Accuracy also decreased between Known and Unknown levels of the Content factor. (B) Response times were significantly increased for the Unknown compared to the Known Content factor.

As expected, behavioral results showed that the task was more demanding when no cue was available (Fig. 2A). Specifically, a repeated-measures ANOVA showed a significant accuracy decrease during the Uncued compared to the Cued condition (69.33% vs. 73.75% correct responses for the Known-Content condition, and 70.58% vs. 76.33% for the Unknown-Content condition, F_1,24_= 23.097,*p*< 0.001). A post-hoc t-test confirmed this difference for the effect of Cueing in both the Known (t_24_= 2.212,*p*< 0.0367) and the Unknown conditions (t_24_= 3.699,*p*< 0.001). There was also a significant difference in accuracy across Known and Unknown levels of the Content factor (F_1,24_= 10.16,*p*= 0.004) but no interaction between Cueing and Content (F_1,24_= 0.516,*p*= 0.516).

There was no significant difference in response times for Cueing (F_1,24_= 0.024,*p*= 0.879), while response times differed for the Content factor (F_1,24_= 12.564,*p*= 0.002). No interaction was observed between factors (F_1,24_= 0.000,*p*= 0.995) (Fig. 2B). The matched reaction times across cued and uncued trials indicate that the benefit of the cue may largely dissipate by the time the second stimulus is presented, thus not influencing response speed upon processing of the latter. Contrarily, increased readiness for the first stimulus afforded by the cue allows for more efficient processing of this stimulus, increasing accuracy of its comparison against the second stimulus of the pair. This observation points to the preparatory (Intertrial) epoch as a behaviorally particularly consequential period in this paradigm. For completeness,Supplementary Figure 1shows that ITI length had no systematic impact on accuracy. In the following, we will investigate the network-level neural processes engaged to meet the increased cognitive demands of the Uncued condition during this period of endogenous preparedness.

### 3.2 Brain activations

**Fig. 3.**
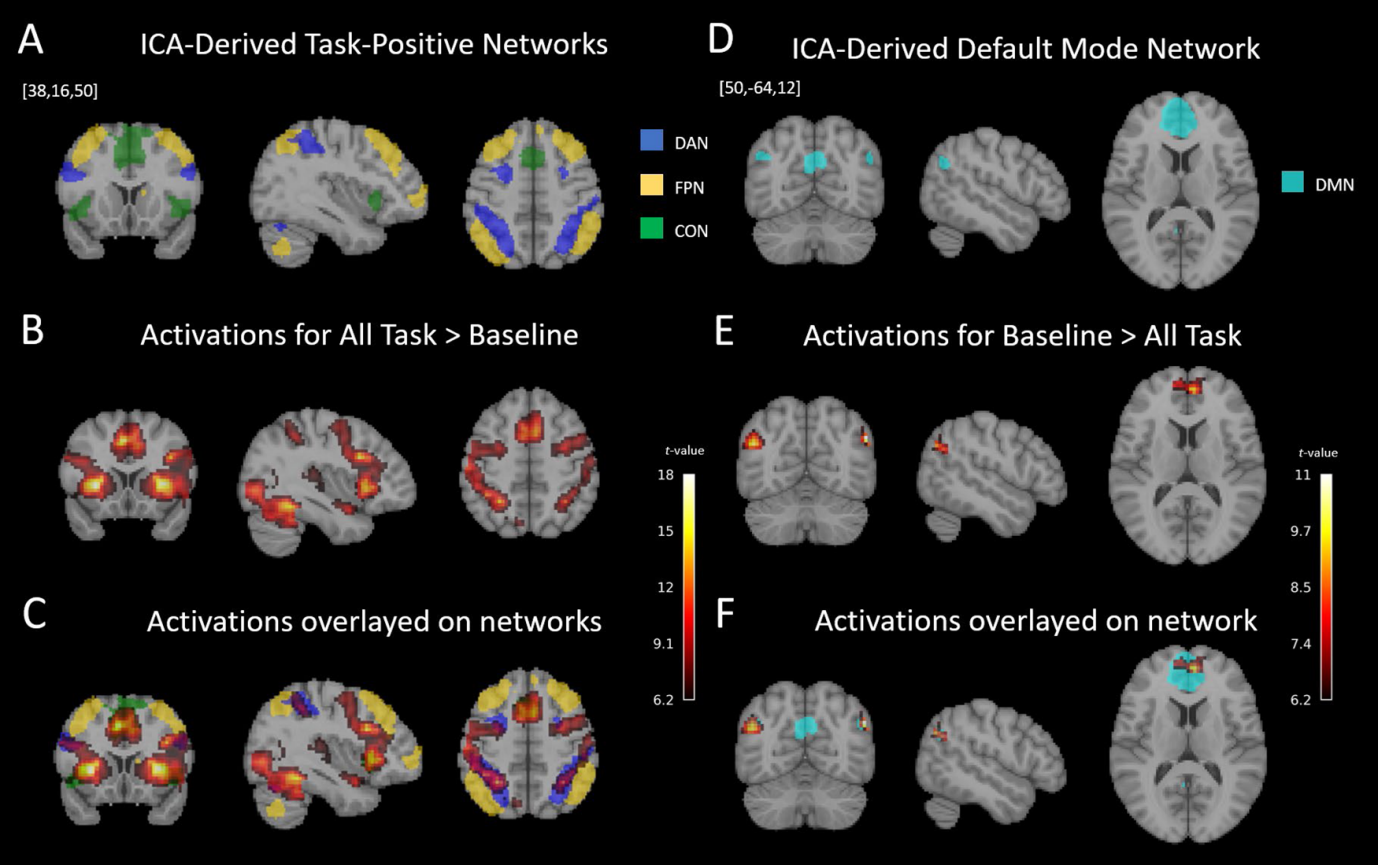
Task-related activations and deactivations overlaid on canonical ICN maps. (A) ICNs from an ICA-based atlas of an independent subject group (Shirer et al., 2012) are shown for the well-known task-positive cognitive control networks, the FPN, DAN, and CON. (B) Activations evoked by all task conditions during the task blocks (irrespective of content or cueing) compared to the implicit baseline (all inter-block intervals) (voxel-wise FWE-corrected,*p*< 0.05). (C) The general effect from (B) overlaid with the ICA-derived network maps from (A) for direct comparison, showing considerable overlap with the DAN and CON, and to a lesser extent the right FPN (cf.Supplementary Table 1). (D) ICA-derived connectivity map for the DMN. (E) The inverse of the general effect of task, that is, deactivations compared to implicit baseline (voxel-wise FWE-corrected,*p*< 0.05). (F) The inverse of the general effect from (E) overlayed with the ICA-derived DMN for visualization. The task deactivates DMN regions as expected. Unthresholded t-maps are provided athttps://neurovault.org/collections/16122/.

Prior to investigating differential network processes across task conditions, we confirmed that activation and deactivation of large-scale ICNs commonly implicated in cognitive control-demanding tasks are observed in our task (all Intertrial and Trial regressors combined contrasted with implicit baseline inter-block intervals and vice versa;Fig. 3). As expected, there was overlap with most regions of the ICA-derived ICNs. For a full list of overlapping regions with their peak activations, please refer toSupplementary Table 1.

**Fig. 4.**
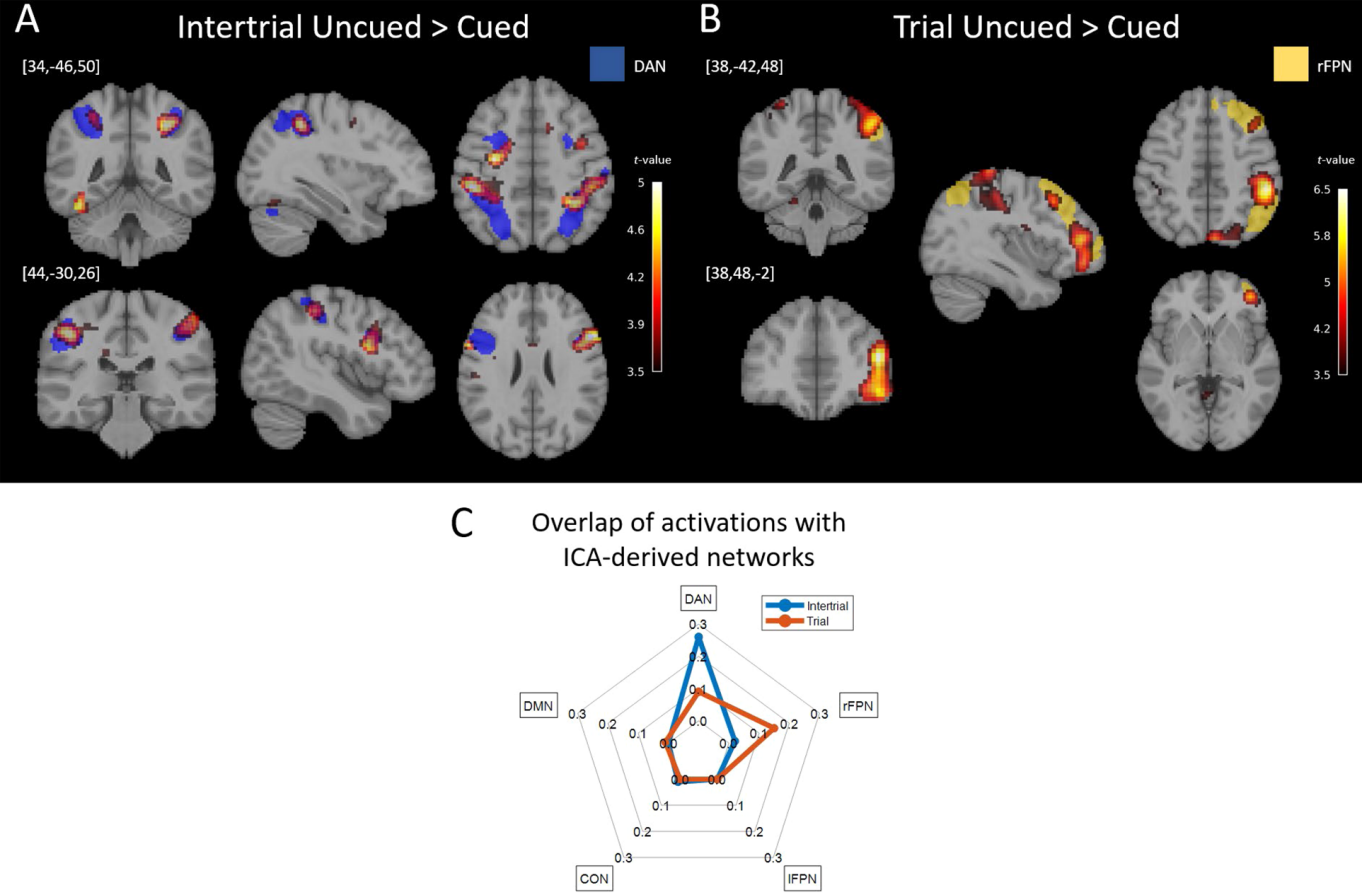
Network activations in the Intertrial and Trial periods. All heat maps highlight Uncued>Cued contrasts. (A) Significant activations during the Intertrial period in the Uncued>Cued condition were found in the left IPS and right FEF (cluster-level FWE-corrected*p*< 0.05). Activations are visualized at voxel-wise*p*< 0.001 uncorrected to emphasize the spatial specificity even under less stringent thresholding. The resting-state ICA-derived DAN (blue) is overlaid to visualize the spatial overlap. (B) Significant activations during the Trial period in the Uncued>Cued condition were found in the right IPC and right vlPFC (cluster-level FWE-corrected*p*< 0.05). Again, activations are presented at voxel-wise*p*< 0.001 uncorrected for visualization purposes. The ICA-derived rFPN (red) is overlaid to demonstrate the spatial overlap. (C) A radar plot quantifies the proportions of the activation maps from A) and B), respectively, that overlap with each of the ICA-derived cognitive control ICNs. ICNs engaged differently in response to the absence of cues, with the DAN showing significantly increased activity in the Intertrial period when no external information was available, and the rFPN showing the most prominent activation in the Trial period. Unthresholded t-maps are provided athttps://neurovault.org/collections/16122/.

To investigate the effects of Cueing, Content, and the interaction between the two, activation contrasts were constructed separately for the Intertrial and the Trial periods. In the Intertrial period, significant activations were found for the main effect of Cueing, with higher activity in the Uncued condition in the left Intraparietal Sulcus (left IPS: cluster-level FWE-corrected*p*= 0.028,*k*= 369, peak voxel location = [-45,-34,43] and*z-score*= 4.06), and the right posterior Inferior Frontal Gyrus (right IFG, cluster-level FWE-corrected*p*= 0.045, voxel extent*k*= 299, peak voxel location = [63,17,22] and*z-score*= 4.04). These regions overlap with the ICA-derived DAN (26.28% of the task activation map overlapped with the ICA map,Fig. 4C), suggesting that the DAN plays a critical role in the maintenance of preparatory cognitive control while waiting for an upcoming stimulus under temporal uncertainty. No activation increases were observed for the reverse contrast Cued > Uncued in the Intertrial period. No significant main effect of Content or interaction between Cueing and Content was found in the Intertrial period.

During the Trial period, significant activations were found in the Uncued > Cued contrast in the right Inferior Parietal Cortex (right IPC: cluster-level FWE-corrected*p*= 0.001,*k*= 623, peak voxel location = [51,-37,49] and*z-score*= 4.9), and right ventrolateral Prefrontal Cortex (vlPFC: cluster-level FWE-corrected*p*= 0.008,*k*= 373, peak voxel location = [42,47,13] and*z-score*= 4.59). These regions overlap with the ICA-derived rFPN (16.26% overlap,Fig. 4C), suggesting that the rFPN plays a role in regulating processing*during*stimulus and response. Additionally, activation was found in a visual cluster outside of all control-related networks (dorsal cuneus/precuneus cortex,*p*= 0.008,*k*= 373, peak voxel location = [6,-85,40] and*z-score*= 4.24) (Fig. 4B). For the reverse contrast of Cued > Uncued in the Trial period, we observed activity only in early auditory cortex reflecting the sensory response to the warning signal (cluster-level FWE-corrected*p*= 0.03,*k*= 249, peak voxel location = [69,-22,13] and*z-score*= 4.62). We found an expected main effect of Content driven by the sensory modality of the stimuli, such that the Known condition (i.e., faces only) broadly activated visual cortex more so than the Unknown condition (i.e., mixture of visual and auditory stimuli), while the latter showed stronger activity predominantly across broad auditory cortices (seeSupplementary Text 1). The interaction between Cueing and Content in the Trial period showed significant activations in the right superior cerebellar area (cluster-level FWE-corrected*p*= 0.002,*k*= 470, peak voxel location = [30,-43,-23] and*z-score*= 5.21) and a right superior parietal region (cluster-level FWE-corrected*p*= 0.031,*k*= 215, peak voxel location = [45,-31,46] and*z-score*= 4.09).

While the CON was highly active during the entire task (i.e., both Intertrial and Trial) (Fig. 3), it did not show any*difference*in activation levels across the conditions, in line with the proposed function of this network in tonic alertness (Sadaghiani & D’Esposito, 2015;Sadaghiani et al., 2010) and sustained task-set maintenance (Dosenbach et al., 2006).

### 3.3 Connectivity

Next, we asked whether the control-related ICNs that were activated in the Cueing contrast interacted with other brain regions, including other ICNs, to accomplish their top-down modulatory function. Specifically, given that the absence of a cue increased*activation*predominantly in the DAN and rFPN in Intertrial and Trial periods respectively, we investigated whether these networks show modulated*connectivity*to other brain areas during increased cognitive control demands in Uncued blocks. To this end, two PPI connectivity models (Friston et al., 1997) focusing on Cueing were generated for each subject for Intertrial and Trial periods, respectively. For these analyses, Known and Unknown stimulus blocks (i.e., Content factor) were combined given that the activation effects of Cueing did not depend on Content in the previous analyses.

**Fig. 5.**
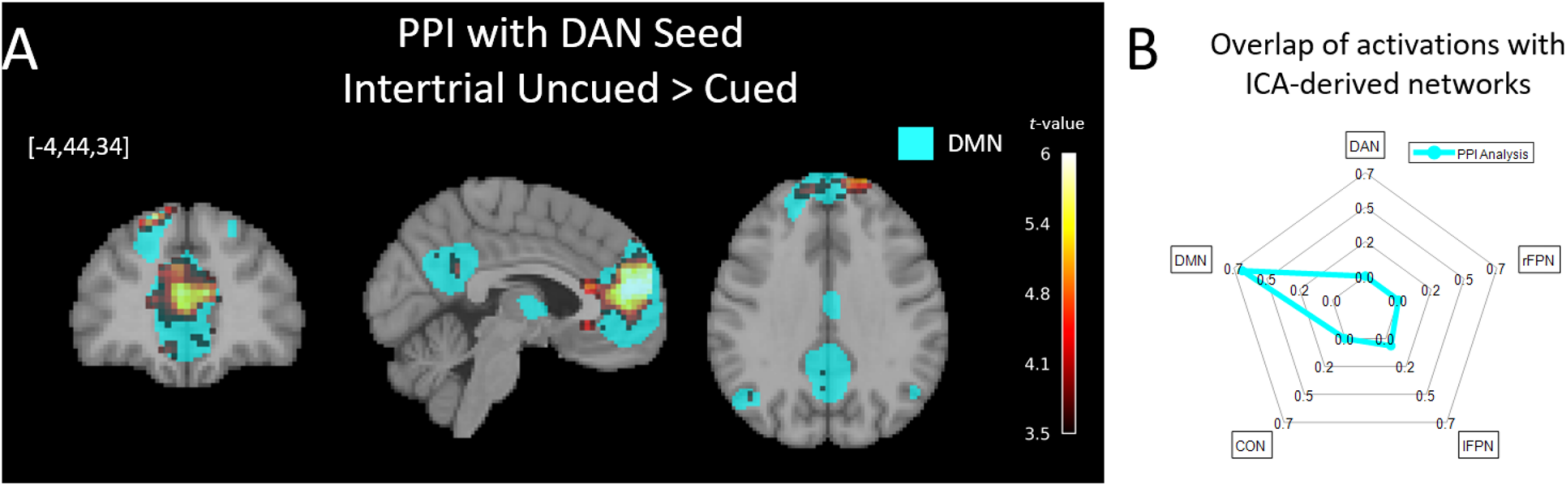
Voxel-wise PPI using the DAN as the seed region. Subject-specific activations (Uncued > Cued contrast) within the DAN ICN constituted the seed volume. For this seed, we assessed changes in connectivity to all other voxels in the brain as a function of the Uncued > Cued contrast during the Intertrial period (collapsed over both Content conditions). This contrast reflects heightened demands on endogenous preparatory control. (A) Significant increases in co-activation were found between the DAN and the medial PFC (cluster-level FWE-corrected*p*< 0.05). The medial PFC is a core component of the ICA-derived DMN (overlaid in cyan for visualization purposes). Activations in this figure are presented at*p*< 0.001 uncorrected to show anatomical specificity to the DMN even when the stringent correction is loosened. (B) A radar plot quantifies the proportion of the activation map from A) that overlaps with each of the ICA-derived cognitive control ICNs. The dominance of the DMN as the volume with the strongest context-dependent co-activation with the DAN is evident. Unthresholded t-maps are providedhttps://neurovault.org/collections/16122/.

The first model utilized the DAN as a seed and assessed how this network’s connectivity is modulated as a function of Cueing in the Intertrial period. More specifically, the seed comprised all voxels within the ICA-derived DAN mask that showed differential activity for Uncued versus Cued Intertrial periods (cf.Fig. 4A) for each individual subject. The context-dependent contrast was designated as the Uncued > Cued Intertrial contrast. We found that when cues were absent, the connectivity of DAN to the medial Prefrontal Cortex became more positive (mPFC: cluster-level FWE-corrected*p*< 0.001,*k*= 1162, peak voxel location = [-12,56,13] and*z-score*= 5.51) (Fig. 5). The mPFC region overlapped with the DMN (66.93% overlap, cf.Fig. 5B). More positive connectivity between DAN and DMN regions could either reflect increased positive connectivity or reduced anticorrelation, but may be interpreted as increased cooperation in either case (Fornito et al., 2012).

For comparison, the second model used an equivalent approach with the rFPN as a seed (voxels with differential activity in Uncued > Cued contrast during the Trial, cf.Fig. 4B) and investigated how its*connectivity*changes by Cueing condition during the Trial period. The context-dependent contrast was designated as the Uncued > Cued Trial contrast. No significant modulation of connectivity was observed.

So far, we assessed connectivity specifically for the ICNs already observed to be involved in Cueing-dependent modulation based on activation (the DAN and rFPN). However, it is conceivable that changes in connectivity occur between pairs of regions even in the absence of change in activation of either region. Therefore, we next sought to explore connectivity in an anatomically comprehensive manner beyond the hypothesis-driven focus on the DAN and rFPN seed volumes. In this exploratory analysis, we investigated connectivity among all individual regions (39 total) of*all*ICA-derived ICNs of interest—the CON, DMN, lFPN, rFPN, and DAN. Considering a possible divergence of connectivity from activity, one might further compare connectivity across Known and Unknown conditions even though these conditions did not evoke differences in activity. Therefore, beyond the spatially inclusive approach, we also widened the conditions back to a comprehensive 2x2 design for a maximally comprehensive approach. For every subject and separately for Intertrial and Trial periods, this approach generated a 39x39 connectivity matrix for each of the four experimental conditions (i.e., Cued Known, Uncued Known, Cued Unknown, Uncued Unknown). Regions along the matrix rows represent (PPI) connectivity seeds, and regions along the columns represent the regions for which Cueing-dependent connectivity modulation to the seed is assessed. To test whether the main factors of Cueing and Content, and their interaction, affect connectivity, we then ran a 2x2 ANOVA at each connection (i.e., each matrix element, multiple comparisons-corrected using a cluster-based approach in graph space).

**Fig. 6.**
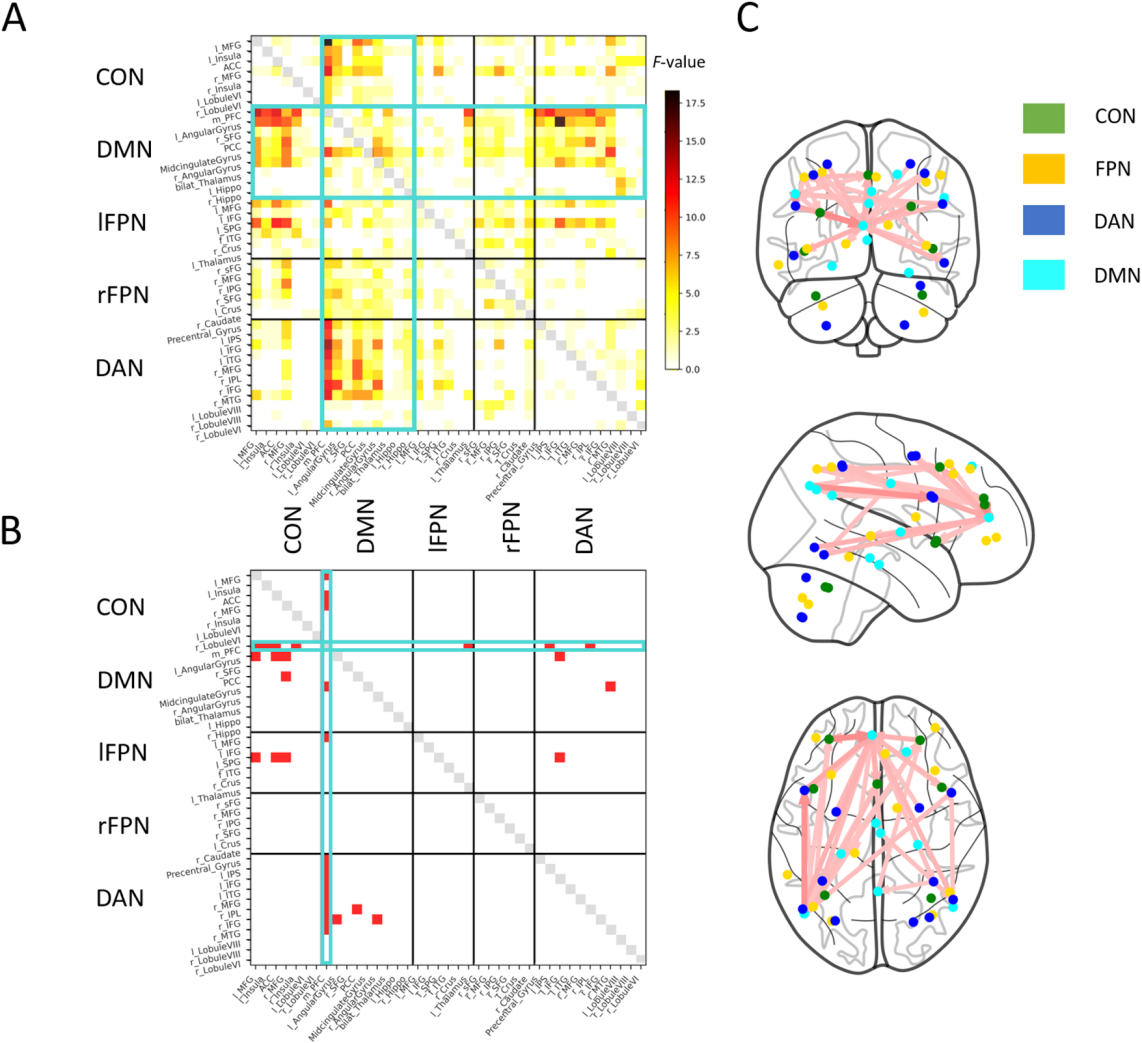
Anatomically comprehensive network-based comparisons of connectivity as a function of Cueing during the Intertrial period. (A) Connectivity matrix showing the F-values of the main effect of Cueing from the 2x2 ANOVA in the Intertrial interval. Each row represents the seed region used in the respective PPI analysis (a separate PPI calculation per seed), while each column is the ROI for which the voxel-wise PPI parameter estimates were averaged. The teal highlights emphasize the increased activation of the DMN. (B) After thresholding each element (connection) of the matrix at F_(1,24)_>9.55 (*p*< 0.005 uncorrected), we applied directed NBS for cluster-based statistical correction of multiple comparisons in graph space. NBS identified a significant cluster of 33 connections between 21 nodes (*p*= 0.021). Specifically, increased co-activations for uncued (vs. cued) Intertrial periods were observed between DMN and DAN regions (15 connections) as well as between DMN and CON regions (11 connections), with the greatest number of edge connections connecting to the medial PFC of the DMN (18 connections, highlighted in teal). (C) The significant cluster from 6b is shown in anatomical space over a glass brain. The medial PFC constitutes a hub in the cluster. Nodes are color-coded for the ICN they are part of.

This exploratory analysis found a significant main effect of Cueing on connectivity in a cluster of interconnected connections during the Intertrial period (Fig. 6A). All edges of the cluster had stronger connectivity in the Uncued Intertrial period (when endogenous preparatory control demands are higher) compared to the Cued condition. This cluster comprised 33 edges connecting 21 nodes (NBS permutation-based*p*= 0.021,Fig. 6B). All but 4 of the 33 cluster edges involved the DMN, with the medial PFC node of the DMN playing a critical role as a hub of the cluster connected to 15 of the edges. A substantial proportion (18) of the edges connected the nodes of the DMN and DAN in line with our DAN-based analysis (cf.Fig. 5). Specifically, the medial PFC was connected to all DAN nodes except the Cerebellar nodes (Lobules VI and VIII). Beyond medial PFC connections, other edges connected the DMN’s left Angular Gyrus to DAN’s Inferior Frontal Gyrus. Further, the significant cluster prominently contained 11 connections between the DMN and the CON. In particular, the medial PFC showed strong connectivity modulation to the left MFG and both the medial PFC and left Angular Gyrus nodes to the ACC. Significant edges connecting to nodes of the lFPN were sparser and included connections between the left SPG to the medial PFC and ACC. We found no main effect of Content or a Cueing-Content interaction on connectivity during the Intertrial period.

In contrast to the Intertrial period, no significant connectivity modulation was observed for any factor or the interaction of factors during the Trial period, suggesting that within our experiment, inter-network interactions are primarily observed in endogenous preparatory processes.

## 4 Discussion

The goal of this study was to delineate the role of canonical control-related networks and their cross-network interactions in facilitating endogenously maintained, preparatory cognitive control. Behavioral outcomes confirmed that the Uncued condition imposed heavier demands on cognitive control, which—during the Intertrial period—had to be met endogenously in anticipation of stimulus onset. We observed increased activity of the DAN associated with preparatory control during the Intertrial period, whereas the right-hemispheric FPN was more strongly engaged in control over the processing of external information during the Trial itself. Most notably, endogenous control was uniquely associated with increased cross-network interactions when no cues were present to rely on. Specifically, though the DMN did not change*activity*levels across task conditions, it showed more positive*connectivity*with the DAN and (to a smaller degree) the CON during the maintenance of endogenously driven preparatory control.

Our experimental approach to isolate endogenous preparation in the*absence*of incoming external information revealed a role for cross-ICN interactions specifically in this internally maintained control process. In the volume-of-interest based connectivity analyses using the DAN and FPN as seeds, respectively (Fig. 5), increased inter-ICN interactions occurred only during the preparatory Intertrial period and were not observed during the Trial itself. Further, this effect was not impacted by the Content factor, suggesting that the preparatory control process isolated in our experiment may constitute general rather than domain-specific readiness. Specifically, the DMN showed increased coactivations with the DAN during endogenous maintenance of readiness. Even when a comprehensive set of regions from all ICNs were used as seeds (Fig. 6), the increased cross-ICN interactions during the Intertrial period were dominated by the DMN; The DMN’s mPFC node was the most densely connected hub of the cross-ICN connectivity change. This finding may shed light on the reasons for the specificity of results to the endogenous control condition. In particular, while the DMN is known to deactivate in most task and stimulation conditions compared to baseline (Fox et al., 2005,^2009^, as confirmed in our task, cf.Fig. 3B-F),*increased*activity is observed in certain conditions. Such increase most notably occurs during cognitive processes that harness internal information, such as episodic memory retrieval, mental time travel, self-referential thought and introspection, as well as processes that involve taking the perspective of others (Buckner et al., 2008). Perhaps then, the DMN is also implicated in*internally*harnessing resources for cognitive control, that is, when control cannot be aided by external events. A related interpretation could be that during endogenously maintained task engagement, the DMN assesses the subject’s internal state in a goal-directed manner, specifically by interacting with task-positive control networks (foremost the DAN) to determine that the latter networks are “on task.”

Our findings extend a growing body of observations that highlight the role of increased DMN connectivity in cognitively demanding tasks, such as memory recall and autobiographical goal-directed planning (Fornito et al., 2012;Spreng et al., 2010), matrix reasoning, and working memory (Hearne et al., 2017;Murphy et al., 2020;Vatansever et al., 2015). However, for methodological reasons (Amico et al., 2017;Fox et al., 2009), it is challenging to determine whether the increase in DMN’s connectivity to other ICNs reflects positive correlations or the dampening of anticorrelations. Both cases, however, represent a more positive inter-ICN interaction that has been interpreted as cooperation (Fornito et al., 2012), which counters the deactivation-based view that the DMN is responsible for a “default” mode of brain function that gives way to other functions when the brain engages in goal-directed processing. Most of the previous studies that report increased connectivity with the DMN have found interactions between the DMN and FPN (Chen et al., 2013;Fornito et al., 2012;Spreng et al., 2010), but not between the DMN and the DAN (and rarely the CON) as is observed in the current study. Our unique approach to investigating preparatory cognitive control in the absence of external information may explain this difference from prior literature in which even internally-oriented processes were studied using external prompts (Fornito et al., 2012;Spreng et al., 2010). In a purely endogenous context, more positive DMN-DAN and DMN-CON connectivity may reflect interactions among internally driven cognitive processes of the DMN and goal-oriented modulatory functions of the DAN and the CON.

Whereas*connectivity*between the DMN and DAN as well as between the DMN and CON became more positive as a function of heightened demands on endogenously driven preparatory control, only the DAN showed increased*activity*in this contrast. Conversely, the CON was strongly activated, and the DMN deactivated,*throughout*the experiment without modulation across task conditions (cf.Fig. 3). These findings highlight the functional dissociation between activation and connectivity serving cognitive control. The continuously high level of CON activity is consistent with its suggested role in tonic alertness (Sadaghiani & D’Esposito, 2015;Sadaghiani & Kleinschmidt, 2016) and*sustained*maintenance of task set (Dosenbach et al., 2006) (and confirms that the respective regions are not related to transient salience processing known for the Salience Network). In contrast, the condition-dependent modulation of DAN activity is consistent with the suggested phasic nature of its function (Sadaghiani & Kleinschmidt, 2016).

While both the DAN and the FPN are implicated in phasic control (Sadaghiani & Kleinschmidt, 2016), we found a functional dissociation between the two networks. Specifically, the pattern of DAN and FPN activity differed during the increased cognitive demands of the cue-free condition. Increased DAN activity was observed during the pre-trial preparatory period whereas increased FPN activity was observed during cue-free blocks once incoming information became available. These observations suggest that the FPN plays a central role in modulation of stimulus-related information during the trial. In summary, while previous work often finds that the FPN and DAN coactivate under cognitive control demands (e.g.,Dosenbach et al., 2007;Sadaghiani & D’Esposito, 2015), when endogenously maintained control is experimentally separated from control initiated by or acting upon external information, the roles of the DAN and FPN diverge.

The increase in FPN activation during the trial was lateralized to the right hemisphere. The intrinsic architecture of ICNs under task-free conditions indicates that the FPN may be functionally more strongly lateralized than other ICNs, since it more readily splits into separate right- and left-hemispheric FPN components in data-driven decompositions (e.g.,Smith et al., 2009;Varoquaux et al., 2010, and the ICN atlas used in the current study (Shirer et al., 2012)). This split indicates that connectivity is substantially stronger among FPN nodes of the*same*hemisphere than*across*hemispheres, perhaps reflecting partially divergent functional specialization in each hemisphere. Our right-lateralized findings may be linked to right-hemispheric dominance during modulatory control over incoming information observed in prior studies (Corbetta et al., 2008;Shulman et al., 2010;Spagna et al., 2020;Sturm & Willmes, 2001), and are in line with cognitive control aberrations such as spatial neglect and reduced vigilance upon damage specifically to the right hemisphere (Corbetta & Shulman, 2011;Karnath & Rorden, 2012;Weinberg & Harper, 1993).

We acknowledge that the sample size in the current study (N = 25), which was based on effect sizes of our prior work (Sadaghiani & D’Esposito, 2015), is relatively small compared to many other neuroimaging studies. We note that studies of the current type, which use carefully designed cognitive tasks tuned to maximize within-subject contrasts of interest, and which seek to show the consistency of these within-subjects effects over the group, often require substantially smaller sample sizes than other study designs such as cross-subject association studies or those that rely on smaller within-subject effects (Gratton et al., 2022).

In summary, by studying preparatory processes independently of external information, we discovered a role of DAN activation and of DMN-centered cross-ICN cooperation in endogenously driven preparatory control. The cross-ICN interactions suggest a functionally important interplay between the DMN and networks more traditionally associated with cognitive control (DAN, CON) in endogenously maintained preparation. The central position of mPFC in this interplay may extend the DMN’s known role in internally-oriented processing to the domain of cognitive control when control must be maintained endogenously.

## Supplementary Material

Supplementary Material

## Data Availability

Code can be found athttps://github.com/connectlab/endog_prep_control.
