# Supplementary material for: Endogenous preparatory control is associated with increased interaction between default mode and dorsal attention networks
Source: Imaging Neurosci (Camb). 2024 Apr 8;2:imag-2-00124. doi: 10.1162/imag_a_00124 (PMC12247561; doi:10.1162/imag_a_00124)

SUPPLEMENTARY MATERIALS

Supplementary Table 1. T values for individual nodes in the contrast of all regressors > implicit baseline.

Network	Node	X	Y	Z	Peak *Z*-Score
All Regressors > Implicit Baseline					
CON	L Insula	-33	20	4	>8
	R Insula	33	20	4	>8
	Bilateral dorsal Anterior Cingulate Cortex	-3	17	43	7.72
	L Cerebellar Lobule VI	-33	-52	-20	6.98
rFPN	L Cerebellar Lobule VI	-30	-58	-32	6.73
	R Inferior Frontal Sulcus/Gyrus	48	14	34	6.31
	R Caudate	12	11	7	6.37
DAN	L posterior Inferior Frontal Gyrus	-45	8	28	7.47
	L posterior Inferior Frontal Gyrus	45	14	31	7.02
	L Intraparietal Sulcus	-27	-52	49	7.3
	R Intraparietal Sulcus	33	-52	49	6.81
	R Inferior Temporal Gyrus	48	-61	-14	6.6
	L Inferior Temporal Gyrus	-45	-67	-8	6.71
	L Frontal Eye Field	-30	-4	52	6.36
	R Frontal Eye Field	30	-1	52	6
	R Cerebellar Lobule VI	39	-67	-26	6.38
DMN	Bilateral Thalamus	6	-16	4	7.55
	L Hippocampus	-18	-31	1	6.96
All Regressors < Implicit Baseline					
DMN	Bilateral medial PrefrontalCortex	6	53	13	6.06
	L Angular Gyrus	-48	-64	31	6.06
	R Angular Gyrus	51	-67	34	5.88
	Bilateral subgenual Anterior Cingulate Cortex	6	35	-8	5.74

All regions significant corrected at voxel-wise level using FWE. Because FWE-corrected voxels extended contiguously over neighboring regions of multiple different ICNs, peak voxels were selected after masking the activation map with the ICA-based ICNs (Which does not affect the voxel-wise statistics).

Supplementary Text 1.

During the Trial period, we found expected activations across the Content conditions in line with the difference in sensory modality across these conditions (visual-only in case of Known vs. auditory *and* visual in case of Unknown). Specifically, for the contrast of Known > Unknown content significant activations were found broadly across bilateral visual cortices including the fusiform area (cluster-level FWE-corrected p<0.001, k=4716, peak voxel location=[-6,-91,-11] and *z-score*=6.70), inferior temporal gyrus (cluster-level FWE -corrected p=0.007, k=285, peak voxel location=[30,-1,-35] and *z-score*=4.09), and the superior frontal sulcus (cluster-level FWE -corrected p=0.032, k=186, peak voxel location=[12,50,49] and *z-score*=3.79).

For the reverse contrast of Unknown > Known content during the Trial period, significant activations were found in the broad bilateral auditory cortex (left: cluster-level FWE-corrected p<0.001, k=2931, peak voxel location=[54,-7,-2] and *z-score*>8.00; right: cluster-level FWE-corrected p<0.001, k=5197, peak voxel location=[-54,-25,10] and *z-score*=7.68), posterior cingulate cortex (cluster-level FWE-corrected p<0.001, k=712, peak voxel location=[-9,-31,37] and *z-score*=5.03), and right inferior cerebellar area (cluster-level FWE-corrected p=0.028, k=193, peak voxel location=[18,-76,-44] and *z-score*=4.78).

Supplementary Figure 1. Average accuracy for each of the four experimental conditions stratified by the length of the intertrial interval.

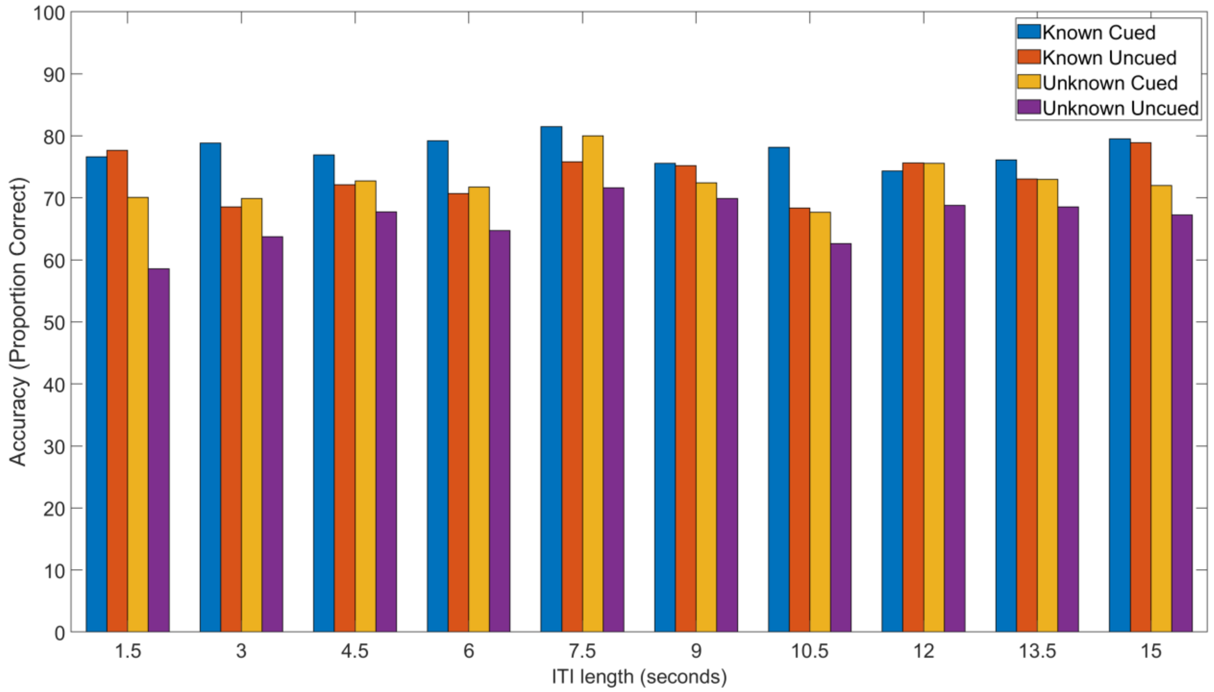

Supplement: Supplementary Material [file imag_a_00124-supp.pdf]
